# Intravenous Immunoglobulin Induces Sustained Hemostatic Responses in IgM Paraprotein-Associated Acquired Von Willebrand Syndrome

**DOI:** 10.7759/cureus.112291

**Published:** 2026-07-08

**Authors:** Mir Alishah, Sibgutallah Imdad, Mubasher Jaffri, Robert Eisner

**Affiliations:** 1 Internal Medicine, University of Chicago (Endeavor Health - NorthShore Hospitals), Evanston, USA; 2 Hematology and Oncology, University of Chicago (Endeavor Health - NorthShore Hospitals), Evanston, USA

**Keywords:** acquired von willebrand disease, igm antibodies, intravenous immunoglobulin (ivig), lymphoma, monoclonal gammopathy

## Abstract

Acquired von Willebrand syndrome (AVWS) associated with monoclonal gammopathy is rare, and current evidence suggests that intravenous immunoglobulin (IVIG) is effective primarily in IgG-mediated disease, with limited benefit in IgM-associated cases. We describe a patient with life-threatening AVWS associated with IgM lambda monoclonal gammopathy secondary to lymphoplasmacytic lymphoma who demonstrated a sustained response to IVIG. A previously healthy woman developed severe mucocutaneous and gastrointestinal bleeding followed by intracranial hemorrhage despite treatment with desmopressin, von Willebrand factor (VWF) concentrates, and antifibrinolytic therapy. Diagnostic evaluation revealed reduced VWF antigen, VWF activity, and factor VIII levels, along with an IgM lambda paraprotein and biopsy-confirmed lymphoplasmacytic lymphoma with *MYD88* mutation. During hospitalization for intracranial hemorrhage, she received VWF replacement and a single dose of IVIG (1 g/kg). VWF antigen increased from baseline values in the 20% range to 93% within 24 hours, peaked at 318% by day 4, and remained elevated at 117% 17 days later, far exceeding the expected duration of replacement therapy alone. This case challenges the prevailing assumption that IVIG lacks efficacy in IgM-associated AVWS and suggests that some patients may derive meaningful clinical benefit, particularly when urgent hemostatic control is required.

## Introduction

Acquired von Willebrand syndrome (AVWS) is a rare bleeding disorder associated with a variety of underlying conditions, including lymphoproliferative disorders and monoclonal gammopathies [1-6]. Intravenous immunoglobulin (IVIG) is an established therapy for AVWS associated with IgG monoclonal paraproteinemia; however, it is generally considered ineffective in IgM-mediated disease [1-5]. We describe a case of IgM lambda monoclonal gammopathy-associated AVWS with a sustained response to IVIG infusion.

## Case presentation

A previously healthy 80-year-old woman with no personal or family history of bleeding developed new-onset mucocutaneous bleeding. Initial laboratory evaluation demonstrated normal prothrombin time, activated partial thromboplastin time, and platelet function testing, but reduced von Willebrand factor (VWF) antigen (31%), VWF activity (<19%), and factor VIII activity (26%-31%), consistent with AVWS. She demonstrated only a transient response to desmopressin (DDAVP). Over time, she developed recurrent severe gastrointestinal bleeding requiring repeated hospitalizations, blood transfusions, and treatment with DDAVP, VWF-containing concentrates, and antifibrinolytic agents. Her clinical course was complicated by hyponatremia and pulmonary edema associated with DDAVP administration, as well as only transient hemostatic responses to VWF replacement therapy. She subsequently developed an intracranial hemorrhage and was transferred to a tertiary care center for neurosurgical management.

Further evaluation identified an IgM lambda monoclonal protein measuring 0.5 g/dL. Bone marrow biopsy demonstrated a CD5-negative, CD10-negative mature B-cell neoplasm comprising approximately 20% of total marrow cellularity. The *MYD88* L252P mutation was confirmed on next-generation sequencing. Thus, the patient was diagnosed with lymphoplasmacytic lymphoma, specifically Waldenström's macroglobulinemia.

During hospitalization for intracranial hemorrhage, she was treated with VWF-containing concentrates and received a single dose of IVIG (1 g/kg). Following IVIG administration, the VWF antigen level increased from baseline values in the 20% range to 93% within 24 hours and peaked at 318% by day 4. Although the early increase may have partially reflected concurrent VWF replacement, a sustained response was observed, with VWF antigen remaining elevated at 117% 17 days after IVIG administration. This prolonged effect contrasts with the short-lived response typically expected following VWF concentrate infusion alone and is more consistent with the known pharmacodynamic profile of IVIG. After initiation of rituximab-based therapy, the patient achieved remission of lymphoplasmacytic lymphoma with normalization of VWF activity, VWF antigen, and factor VIII levels. She has remained clinically stable without recurrent major bleeding. Table 1 highlights the patient's laboratory parameters before and after IVIG administration, along with their clinical status at specific points in time.

**Table 1:** Hemostatic laboratory parameters before and after IVIG VWF: von Willebrand factor; IVIG: intravenous immunoglobulin; NA: not available. The respective lab was not drawn during the period of time where 'NA' is listed.

Timepoint	VWF Ag (%)	VWF Activity (%)	FVIII (%)	Clinical Status
Presentation	31	<19	26–31	Mucocutaneous bleeding
Pre-IVIG	~20	NA	NA	Intracranial hemorrhage
24 h post-IVIG	93	NA	59	Improving
Day 4	318	NA	145	Stable
Day 17	117	107	NA	No recurrent bleeding
Normal range	50–160	50–200	60–150	-

## Discussion

AVWS associated with monoclonal gammopathy is uncommon, and IgM-associated AVWS represents an especially rare subset. Several pathogenic mechanisms have been proposed in AVWS, including autoantibody-mediated accelerated clearance of VWF, adsorption of VWF onto malignant cells, and enhanced proteolysis of VWF multimers [1-6]. The mechanisms underlying differential responses to IVIG in IgG- versus IgM-associated disease remain incompletely understood. In IgG-mediated AVWS, IVIG is thought to inhibit Fc receptor-mediated clearance of VWF-antibody immune complexes and neutralize pathogenic antibodies, resulting in sustained increases in circulating VWF levels [2,3]. In contrast, IgM-associated AVWS may more commonly involve nonimmune mechanisms such as direct adsorption of VWF by paraproteins or malignant cells, potentially explaining the historically limited efficacy of IVIG in this setting [2-6]. Prior studies, including the series by Federici et al., demonstrated that IVIG is frequently effective in IgG-associated AVWS but generally ineffective in patients with IgM monoclonal gammopathies [1]. Similarly, expert reviews and management guidelines have emphasized plasmapheresis and treatment of the underlying clone as preferred therapeutic approaches for IgM-associated disease [2-5].

This case challenges the prevailing assumption that IgM-associated AVWS does not respond to IVIG. In selected cases, immune-mediated mechanisms may contribute more substantially to the pathogenesis of IgM-associated disease than previously appreciated. Importantly, the gradual decline in VWF levels over approximately two weeks closely mirrors the response kinetics described in IgG-mediated AVWS, supporting a biologically meaningful treatment effect rather than transient replacement alone [2,3]. Although IVIG is not routinely recommended for IgM-associated AVWS, it may provide clinically meaningful and sustained hemostatic benefit in acute bleeding settings where rapid control is required.

## Conclusions

In conclusion, we describe a rare case of IgM lambda monoclonal gammopathy-associated AVWS with a sustained laboratory and clinical response to IVIG. This case challenges the prevailing assumption that IVIG has limited efficacy in IgM-mediated disease and suggests that immune-mediated mechanisms may contribute to the pathogenesis in selected patients. Although IVIG is not routinely recommended for IgM-associated AVWS, it may provide meaningful hemostatic benefit in acute bleeding settings when rapid control is required. Further study is needed to better define which patients with IgM-associated AVWS may be most likely to respond to IVIG and to clarify the underlying mechanisms driving treatment response.
